# A Novel Real-Time Threshold Algorithm for Closed-Loop Epilepsy Detection and Stimulation System

**DOI:** 10.3390/s25010033

**Published:** 2024-12-24

**Authors:** Liang-Hung Wang, Zhen-Nan Zhang, Chao-Xin Xie, Hao Jiang, Tao Yang, Qi-Peng Ran, Ming-Hui Fan, I-Chun Kuo, Zne-Jung Lee, Jian-Bo Chen, Tsung-Yi Chen, Shih-Lun Chen, Patricia Angela R. Abu

**Affiliations:** 1 The Department of Microelectronics, College of Physics and Information Engineering, Fuzhou University, Fuzhou 350108, China; eetommy@fzu.edu.cn (L.-H.W.); 221127121@fzu.edu.cn (Z.-N.Z.); rqpfzu@163.com (Q.-P.R.); fan_mh@126.com (M.-H.F.); 2 College of Biological Science and Engineering, Fuzhou University, Fuzhou 350108, China; t20106@fzu.edu.cn; 3 School of Advanced Manufacturing, Fuzhou University, Quanzhou 362200, China; johnlee@fzu.edu.cn; 4 Department of Information and Telecommunications Engineering, Ming Chuan University, Taoyuan 32023, Taiwan; jbchen@mail.mcu.edu.tw; 5 Department of Electronic Engineering, Feng Chia University, Taichung 40724, Taiwan; tsungychen@fcu.edu.tw; 6 The Department of Electronic Engineering, Chung Yuan Christian University, Taoyuan 32023, Taiwan; chrischen@cycu.edu.tw; 7 The Department of Information Systems and Computer Science, Ateneo de Manila University, Quezon City 1108, Philippines; pabu@ateneo.edu

**Keywords:** epilepsy detection, ASIC, closed loop, electrical stimulation, feature extraction

## Abstract

Epilepsy, as a common brain disease, causes great pain and stress to patients around the world. At present, the main treatment methods are drug, surgical, and electrical stimulation therapies. Electrical stimulation has recently emerged as an alternative treatment for reducing symptomatic seizures. This study proposes a novel closed-loop epilepsy detection system and stimulation control chip. A time-domain detection algorithm based on amplitude, slope, line length, and signal energy characteristics is introduced. A new threshold calculation method is proposed; that is, the threshold is updated by means of the mean and standard deviation of four consecutive eigenvalues through parameter combination. Once a seizure is detected, the system begins to control the stimulation of a two-phase pulse current with an amplitude and frequency of 34 μA and 200 Hz, respectively. The system is physically designed on the basis of the UMC 55 nm process and verified by a field programmable gate array verification board. This research is conducted through innovative algorithms to reduce power consumption and the area of the circuit. It can maintain a high accuracy of more than 90% and perform seizure detection every 64 ms. It is expected to provide a new treatment for patients with epilepsy.

## 1. Introduction

Epilepsy is a disease of abnormal brain discharge, which affects people of different ages [1,2]. As a chronic disease, epilepsy itself is not infectious, but its recurrence and sudden onset without warning could still bring considerable pressure to patients. The etiology of epilepsy can be roughly divided into structural, genetic, metabolic, and immune categories. The predisposing factors include prenatal or perinatal brain injury, genetic diseases of brain malformation, severe head injury, and brain infection [3,4]. During seizure in epilepsy, patients suddenly lose control of their bodies, often leading to fracture, bruising, and other conditions, which pose a threat to their safety [5].

Most patients can avoid seizures with the appropriate use of antiepileptic drugs, but for many low-income groups, the treatment options available to them are often limited [6,7]. An increasing number of new antiepileptic drugs have been invented in recent decades, but they still cannot achieve effective effects on all epilepsy treatments [8]. Surgery can be adopted for intractable epilepsy. Surgery mainly achieves the effect of treatment by removing epileptic lesions. The premise of treatment is to accurately locate the lesions. However, the propagation of electrical signals of neurons in the focus area will cause abnormal discharge in the normal area, so accurately locating them is not easy [9]. With the obvious disadvantages of surgery and drug treatment, electrical stimulation therapy has become an effective alternative intervention. Herrera et al. [10] reduced the frequency of seizures by more than 50% in two patients with brain stimulation treatment, with no adverse side effects. Koessler et al. [11] successfully alleviated epilepsy symptoms through high-frequency closed-loop stimulation experiments. In recent years, Ahammed et al. [12] put forward an intelligent system, which can quickly detect patients through machine learning and complete rescue tasks in actual scenarios. Frances-Villora et al. [13] proposed a low-delay epilepsy detection assistant system to help doctors quickly identify a large amount of information. Liang et al. [14] proposed a Koopman-MPC framework for real-time closed-loop electrical neuromodulation in epilepsy. These findings confirm the control of seizures by the basal ganglia circuit and the potential for treating epilepsy through electrical stimulation.

At present, the equipment for closed-loop nerve stimulation research is usually relatively large, which is not convenient for patients. The characteristic calculation of epilepsy is often implemented outside the chip, which is not desirable for deep stimulation. On the basis of the disadvantages of the existing design, an efficient time-domain feature detection method is proposed in this paper. The algorithm not only has high accuracy but also a small area and low power consumption after hardware design, thus meeting the requirements for intrusive equipment.

The rest of the paper is organized as follows: Section 2 introduces the overall block diagram and the proposed algorithm. Section 3 describes the circuit design of each module. The simulation results and analysis are shown in Section 4. Section 5 and Section 6 present the discussion and conclusion, respectively.

## 2. Closed-Loop System Architecture

**Figure 1:**
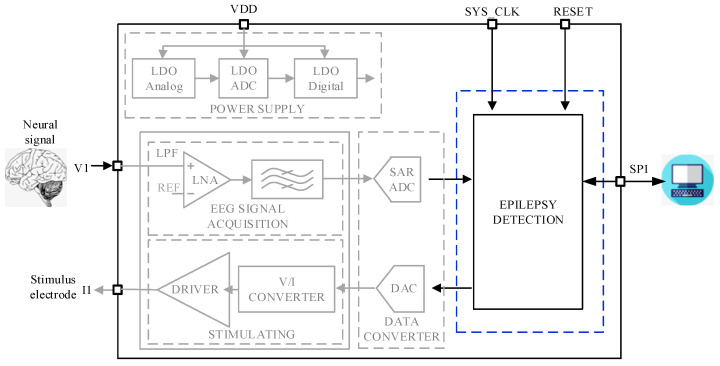
Diagram of the system.

The overall block diagram of the system is shown in Figure 1. The whole system includes five parts: electroencephalogram (EEG) signal acquisition, data converter circuits including analog to digital converter (ADC) and digital to analog converter (DAC), low dropout regulator (LDO), nerve stimulation, and epilepsy detection. In the figure, VDD represents the operating voltage of the chip, and serial peripheral interface (SPI) is the communication interface. A complete closed-loop system can be formed through these five parts.

### 2.1. Analog Front End and Stimulation Circuits

The LDO circuits provide each module with an output voltage that is relatively stable even with changes in the input voltage. The signal acquisition includes a low noise amplifier (LNA) and a low-pass filter (LPF) with a cut-off frequency of 100 Hz. The LNA circuit is designed to filter the noise interference and amplify the weak EEG signal. The effective frequency of EEG is generally not higher than 100 Hz, so the high-frequency noise is filtered by a low-pass filter, and the 50 Hz power frequency interference is filtered by the digital part. A 14-bit successive approximation (SAR) structure ADC was adopted to convert the analog EEG signal to digital data. The final accuracy of a three-stage capacitor structure and digital calibration design could reach 13.8 bits [15]. For the treatment of patients, a bipolar nerve stimulation pulse generator circuit can provide stimulation pulses through a digital signal control H-bridge, which is used to generate a stimulation current for the treatment of patients.

### 2.2. Novel Time Domain Detection Algorithm

Epilepsy detection algorithms often involve three parts, including time domain, time frequency, and nonlinear analyses. Time domain analysis is a straightforward method for examining the temporal features of EEG signals [16,17,18]. In the present study, four signal parameters were selected for epilepsy detection features: signal amplitude (AMP), signal slope (SLP), line length (LL), and energy (POWER) [19,20,21]. The calculation formulas are as follows:

(1) $$LL=\sum_{i=1}^{N-1}abs\left[x\left(i+1\right)-x\left(i\right)\right],$$

(2) $$AMP=\frac{1}{N}\sum_{i=1}^{N}abs\left[x\left(i\right)\right],$$

(3) $$SLP=\frac{Max\left[x\left(i\right)\right]-Min\left[x\left(j\right)\right]}{\left|i-j\right|},$$

(4) $$POWER=\frac{1}{N}\sum_{i=1}^{N}\left[x{\left(i\right)}^{2}\right],$$

where *x*(*i*) represents the EEG signal, and the timescale of *i* is 1 ns.

After different feature parameters were extracted, thresholds were set in accordance with the proposed algorithm, and the results of comparing the threshold with the feature values were used to determine whether an epileptic event occurred.

**Figure 2:**
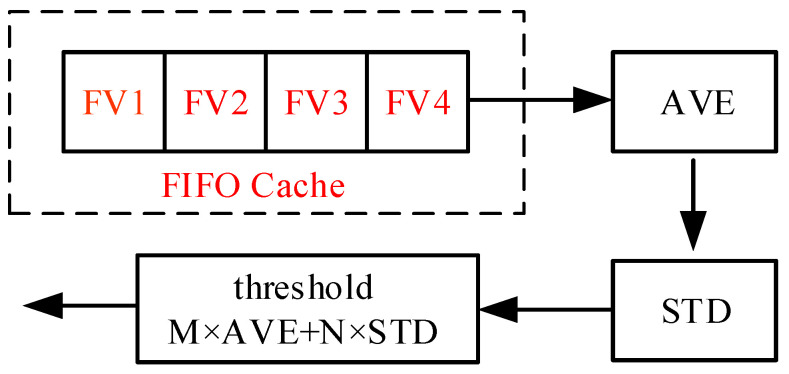
Threshold solution process.

For the time-domain detection algorithm, the result of threshold calculation directly determines the detection effect [22]. This paper presents an effective and easy to implement time domain threshold calculation method. Instead of the traditional complex threshold calculation, it uses the relationship between four adjacent eigenvalues (FV1, FV2, 112 FV3, and FV4) to calculate their average and standard deviation, and then using these to combine parameters which can be used as a new threshold. The eigenvalue and threshold are synchronous and constantly updated. The solution process is shown in Figure 2.

In Figure 2, four eigenvalues of FV1, FV2, FV3, and FV4 are entered in the first input first output (FIFO) cache. If a new eigenvalue comes in, it pushes out the oldest eigenvalue, FV4, as the new FV1, and the remaining features each replace the feature to their right. The mean value (AVE) and standard deviation value (STD) of the four features were obtained by calculation [23] using Equations (5) and (6), where *f*(*i*) represents the feature, as follows:

(5) $$AVE=\frac{1}{4}\sum_{i=1}^{4}f(i),$$

(6) $$STD=\sqrt{\frac{\sum_{i=1}^{4}{\left(f(i)-AVE\right)}^{2}}{4}}$$

According to whether the algorithm can correctly detect epileptic signals and normal EEG signals, the confusion matrix of classification results can be obtained, as shown in Table 1. True positive (TP) indicates that the algorithm predicts the correct number of epilepsy signal samples; true negative (TN) indicates the correct number of normal EEG signal samples; false positive (FP) indicates the number of normal EEG signal samples predicted into epilepsy signal; false negative (FN) indicates the number of epilepsy signal samples predicted into a normal EEG signal.

The programming tool Python was used to simulate the proposed algorithm to obtain the best combination coefficients M and N for each parameter. For example, as shown in Table 2, Table 3, Table 4 and Table 5, through the simulation results, four indices, including the F2 score, recall rate, accuracy, and precision, of SLP can be obtained [24,25,26,27]. The specific definition is as follows:

(7) $$Precision=\frac{TP}{TP+FP}\times 100\%$$

(8) $$Accuracy=\frac{TP+TN}{TP+TN+FP+FN}\times 100\%$$

(9) $$Recall=\frac{TP}{TP+TN}\times 100\%$$

(10) $$F2-SCORE=\frac{5TP}{5TP+4FN+FP}\times 100\%$$

Precision is based on the prediction result, and the proportion of the sample with the prediction as the positive example is predicted correctly. Accuracy represents the proportion of the number of correct predictions to the total number of positive and negative cases. The recall rate is based on actual samples. Among the samples that are positive examples, the proportion of the positive examples that are predicted correctly accounts for the total actual positive examples. The F2 score is a weighted and harmonic average of accuracy and recall, helping to strike the best balance between the two metrics. That is what we focused on in the experiment.

In order to detect seizures more accurately by calculating the threshold in the closed-loop epilepsy detection system. We designed a Python experiment to calculate the optimal threshold for each parameter.

Taking SLP as an example, this experiment conducted 25 experiments on eigenvalues by writing a Python script. Each experiment used a different combination of M and N values to calculate the threshold, and used precision, accuracy, recall, and F2 scores to evaluate the performance of each combination, with the ultimate goal of determining the threshold calculation method that can provide the highest accuracy in epilepsy detection.

It can be seen from the table that under different combinations of m and n values, F2 scores, precision, accuracy, and recall rates of SLP parameters are recorded in detail and displayed in the table. When the parameter of M = 2 and N = 1, the SLP parameter has the highest F2 score and maintains a high accuracy and recall rate. This suggests that this combination of parameters is most effective in identifying seizures. This finding is critical for optimizing the threshold setting of closed-loop epilepsy detection systems, as it not only improves the accuracy of the detection, but also enhances the reliability of the system. It is critical for clinical applications and actual medical device operation.

Through the above simulation, the M and N values corresponding to the best indicators of each parameter can be obtained. The optimal threshold parameters of each parameter finally selected are shown in Table 6. The hardware design is based on these simulation results.

## 3. Circuit Implementation

The main topic of this paper is the digital circuit design for epilepsy detection. The stimulation current and frequency of the whole system are 34 μA and 200 Hz, respectively. The accuracy of epilepsy detection is more than 92%.

### 3.1. Digital Circuit Implementationn

**Figure 3:**
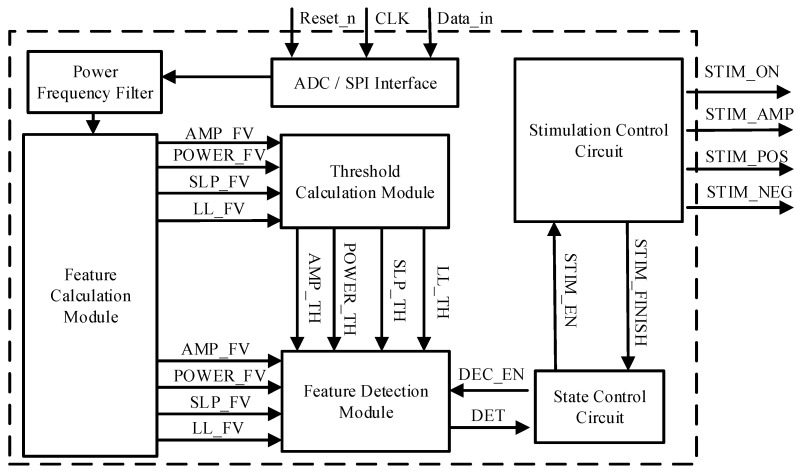
Block diagram of digital circuit.

The block diagram of the digital circuit is shown in Figure 3, including the ADC/SPI interface, feature calculation, threshold calculation, feature detection modules, 50 Hz power frequency filter, state control, and stimulation control circuits. The feature calculation module calculates four feature values in accordance with the quantization results of the ADC or the data transmitted from the SPI interface. After the eigenvalues of calculation are completed, the output signals AMP_FV, POWER_FV, SLP_FV, and LL_FZ need to be transmitted to two modules—the threshold calculation and feature detection modules—at the same time. The threshold calculation module calculates the threshold value in accordance with the corresponding threshold calculation method and transmits signals AMP_TH, POWER_TH, SLP_TH, and LL_TH to the feature detection module. The threshold value is defined in the formula of M × AVE + N × STD.

The feature detection module compares the output results of the feature calculation and threshold calculation modules. After the feature detection module detects the seizure, it transmits the detection signal DET to the state control circuit. The state control circuit starts the stimulation control circuit via the command STIM_EN. Concurrently, the stimulation module starts to control the corresponding stimulation parameters STIM_AMP, STIM_ON, STIM_POS, and STIM_NEG. If the detection time of the stimulation module exceeds the stimulation duration, the signal STIM_EN is generated again. Although the feature detection module is closed at this time, the feature calculation and threshold calculation need to be carried out all the time. If not, some EEG data during stimulation are missed, which could lead to inaccuracies.

### 3.2. ADC/SPI Interface

**Figure 4:**
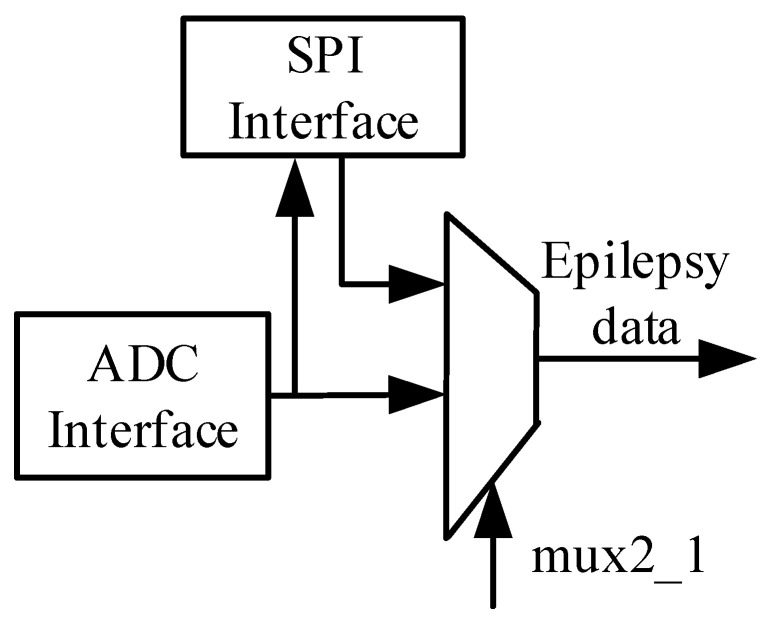
SPI interface data flow.

The data quantified by the ADC circuit are not only sent to the epilepsy detection module but also output to the output port of SPI. Figure 4 shows the data flow of the SPI interface [28,29]. The epilepsy detection circuit receives quantified data from the ADC circuit and EEG signals through the SPI interface simultaneously. The signal mux2_1 is a selective signal that can decide whether to use the signal from the ADC or the signal from the SPI interface.

### 3.3. Status Control Circuit

The initial threshold values were set to zero, indicating that no eigenvalue calculation was performed. After calculations are completed, the TH_FINISH signal increases, indicating that the threshold values are updated, and the digital circuit enters the normal epilepsy detection state.

In this state, it actively monitors signs of seizures based on a constantly updated threshold value. If a seizure is detected, this triggers the DET signal and turns on the STIM_EN signal simultaneously, marking the beginning of the stimulus pattern. Next, the DEC_EN signal is turned off to ensure that the chip shifts to the stimulus process. This shift is key because it represents the chip’s response to a patient’s seizure.

**Figure 5:**
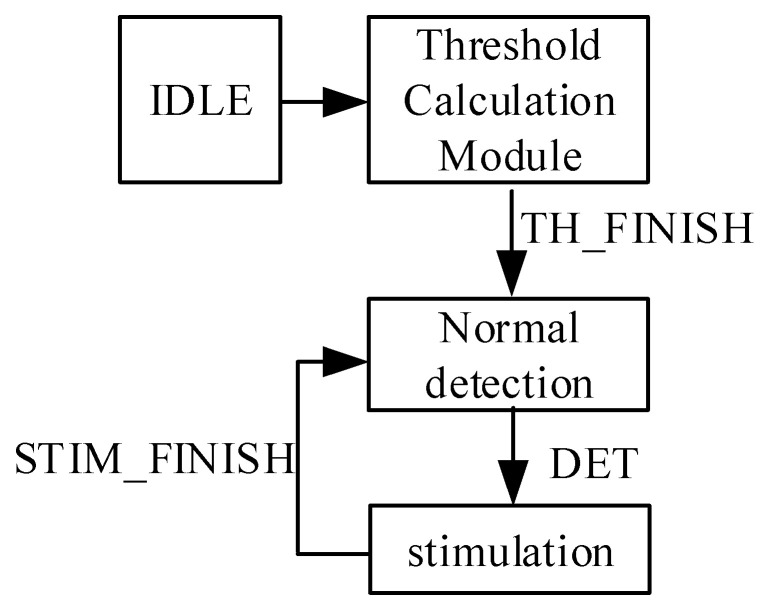
State control flowchart.

Once the stimulus is completed, the system restarts from the threshold calculation state. The system reenables the DEC_EN signal for detection and disables the STIM_EN, thus returning to the normal detection state. As shown in Figure 5, the synchronous transition between states demonstrates the complex design that underpins the chip’s functionality.

### 3.4. Feature Detection Module

**Figure 6:**
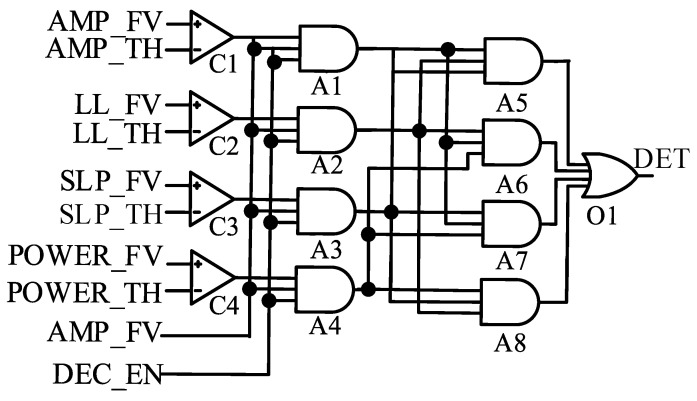
Circuits of the feature detection module, including four comparators (i.e., C1, C2, C3, andC4), and eight AND gates (i.e., A1, A2,…, and A8) and one OR gate (i.e., O1).

The circuit of the feature detection module is shown in Figure 6. It compares the values calculated by the feature calculation module and the threshold calculation module. If the feature value is larger than the threshold value, the output result is set to 1; otherwise, it is set to 0. Simultaneously, the output result of four comparators C1, C2, C3, and C4 can be controlled by two enabling signals AMP_FV and DEC_EN. AMP_FV is the output signal from the feature calculation module. It is maintained until the output of all the features has been completely calculated. DEC_EN is the output signal from the state control circuit, which indicates when epilepsy detection can be started, to avoid epilepsy detection and simultaneous stimulation. Only three of the four eigenvalue signals need to be satisfied to determine the seizure. Thus, eight AND gates and one OR gate were used for control to obtain the final DET signal. If a seizure is detected, the DET signal is 1.

### 3.5. Feature Calculation Module

**Figure 7:**
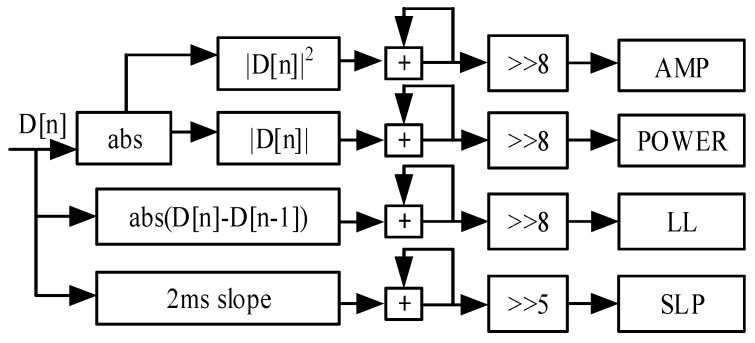
Circuits of the feature calculation module.

The feature calculation module is shown in Figure 7. The amplitude D[n] was employed to calculate the input absolute value and obtain the sum of the amplitude features through 255 accumulations. The final amplitude feature AMP is obtained by dividing 256 by offsetting to the right by 8 bits to obtain. The process to obtain the energy feature POWER is like that for the AMP feature. However, the square of the square needs to be calculated first, and then the cumulative shift is conducted. For the line length feature LL, the absolute value of the difference between two consecutive values needs to be calculated. The feature SLP requires calculation of the slope for 2 ms before the cumulative shift. After the four features are calculated, the resulting signal and feature values are sent to the threshold calculation module for further calculation. This systematic method ensures an accurate analysis of the four AMP, POWER, LL, and SLP signal features.

### 3.6. Threshold Calculation Module

**Figure 8:**
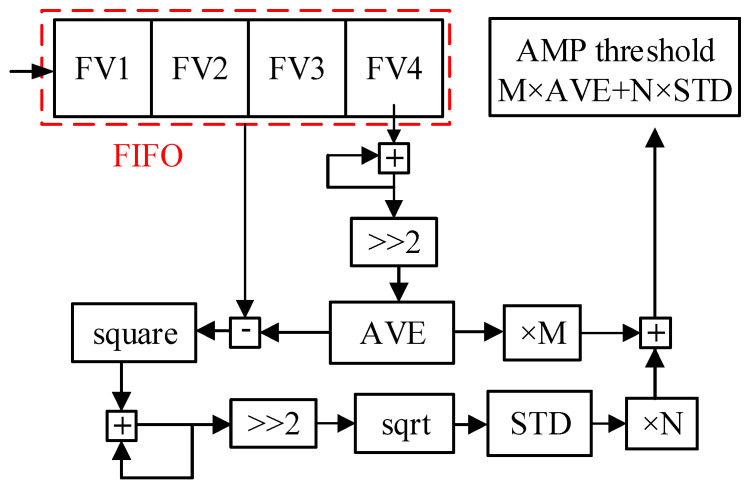
Threshold calculation.

As shown in Figure 8, the threshold calculation methods are consistent, but the threshold parameters M and N are different (as in Table 1), resulting in different final threshold values POWER_TH, AMP_TH, SLP_TH, and LL_TH. Taking the calculation of the amplitude threshold value for AMP_TH as an example, the old eigenvalues FV1, FV2, FV3, and FV4 enter the FIFO cache. When a new eigenvalue comes in and FV4 is removed, the new eigenvalue and the old FV1, FV2, and FV3 become the new FV1, FV2, FV3, and FV4 in FIFO order. The four new eigenvalues are accumulated and summed with Equations (5) and (6) to obtain their mean value (AVE) and standard deviation (STD) [30]. Finally, by using the threshold parameters M and N, AVE and STD are combined to output the amplitude threshold value AMP_TH.

### 3.7. Stimulation Control Circuit

**Figure 9:**
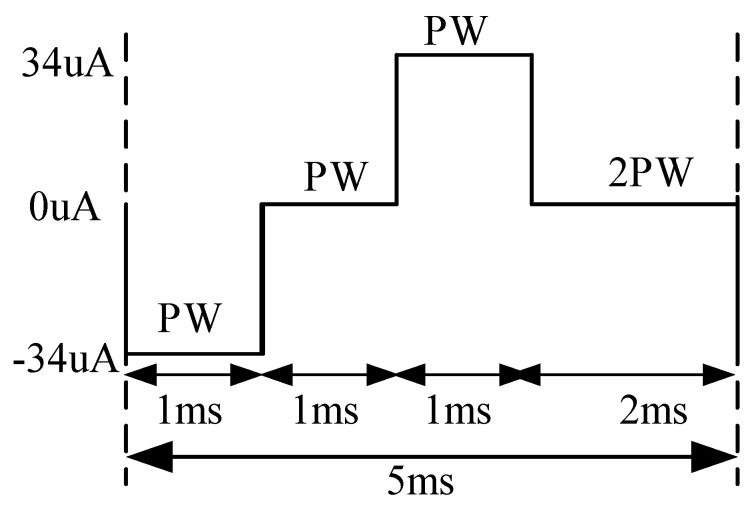
One pulse stimulation cycle.

A 200 Hz stimulation frequency and 34 μA biphasic current pulses were used to avoid charge accumulation and prevent harm to the human body [31,32,33]. The stimulus module initiated by the state control module generates five pulses per stimulation cycle, with each pulse stimulation cycle as depicted in Figure 9. Each positive and negative pulse width (PW) is 1 ms, totaling 5 ms per stimulation period, with corresponding stimulation currents of −34, 0, 34, 0, and 0 μA.

**Figure 10:**
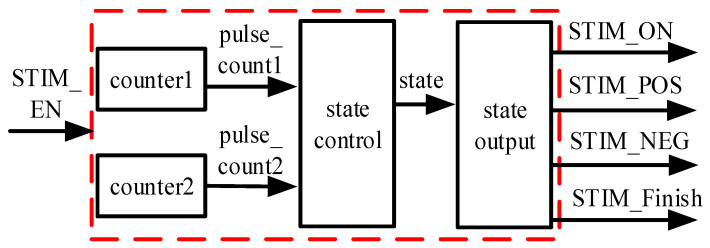
Block diagram design of the stimulation module (The red dotted lines are submodules).

The stimulation control circuit employs two counters; one is used to count each pulse of the system clock, requiring eight counts per pulse with counter 1, and another to track the completion of five pulses with counter 2. The state control circuit controls the state outputs STIM_ON, STIM_POS, STIM_NEG, and STIM_Finish, via the output values of counters 1 and 2. The block diagram of the stimulation control circuit is shown in Figure 10.

## 4. Experiment and Results

### 4.1. FPGA Verification Platform

After layout and wiring, the digital system was ported to a field programmable gate array (FPGA) development board Terasic Altera DE2-115 for functional verification. The resources consumed are 1095 look-up tables (LUTs) and nine digital signal processors (DSPs) after integration on the FPGA, and the power consumption of the FPGA board is 75 mW only. The proposed epilepsy detection module has lower resource utilization and lower power consumption than the previous work in Table 7.

**Figure 11:**
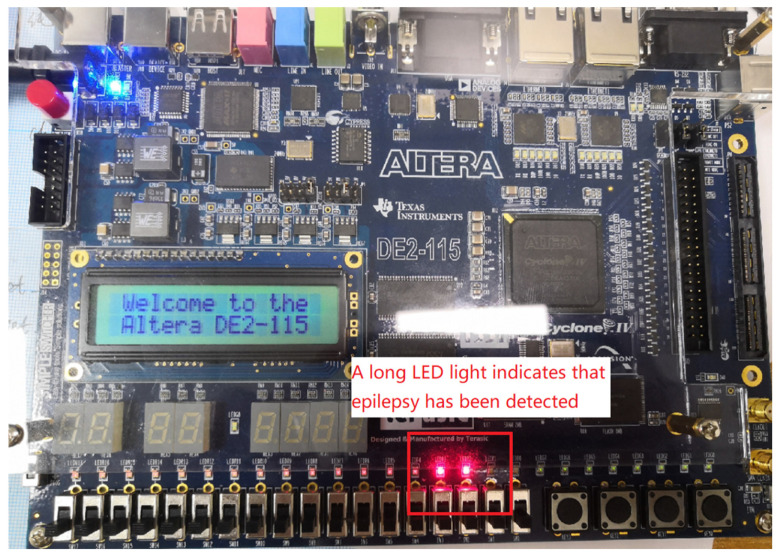
FPGA test results.

For ease of display on the FPGA development board, the output of the stimulus module is connected from pulse to level to the LED light before writ the program onto the FPGA board. As shown in Figure 11, when a seizure is detected, two LED lights on the board light up, indicating that a biphasic stimulating current is generated.

### 4.2. Digital Layout

**Figure 12:**
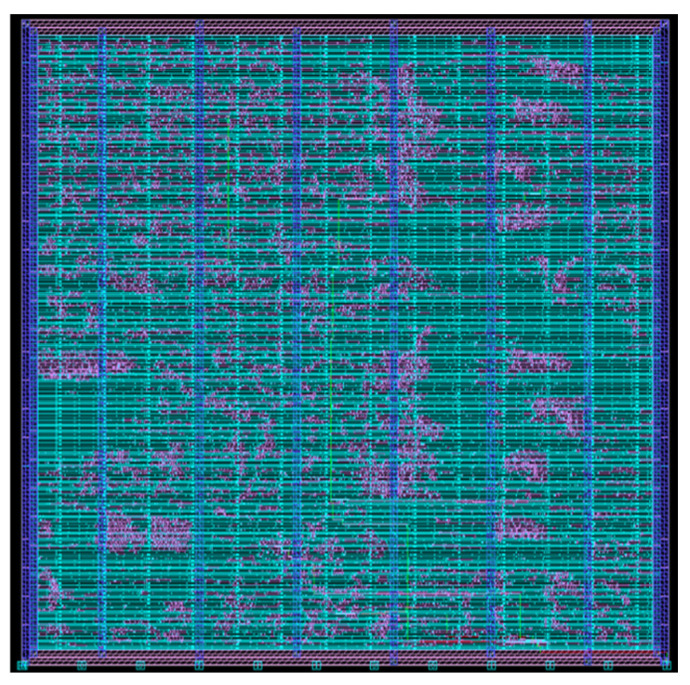
Integral digital layout.

The layout of the epilepsy detection and stimulation digital circuit using application-specific integrated circuits (ASICs) is shown on Figure 12. The circuit is implemented with a UMC 55 nm CMOS general propose process. A comparison between the proposed systems and other seizure detection systems is depicted in Table 8.

Ai et al. [38] based their hardware implementation on the 65 nm process and a convolutional neural network (CNN) algorithm, and found that chip accuracy in assessing seizures could reach 87.9%, with power consumption and chip area 4.45 mW and 2.5 mm^2^, respectively. Lee et al. [39] used a Linear Least Squares (LLS) algorithm and achieved an epilepsy classification accuracy of 88.7% with a chip area of 13.67 mm^2^ and power consumption of 0.992 mW. Zhang et al. [40] used a guided time-channel averaging support vector machine (GTCA-SVM) algorithm. Chip area was only 0.114 mm^2^, power consumption was 0.311 mW, and the accuracy was slightly lower, at 86%. Wen et al. [41] again employed the SVM algorithm, with epilepsy detection chip area 0.98 mm^2^, power consumption, and accuracy being 0.42 mW and 91.86%, respectively. In the proposed system, the chip area and power consumption were, respectively, only 0.0233 mm^2^ and 0.8 μW. The corresponding accuracy of epilepsy detection can reach 94%, and a stimulation current can be generated to provide treatment for patients.

### 4.3. System Simulation

The accuracy of the method in the present study is improved compared with the algorithms used in previous studies [38,39,40,41]. The proposed method is relatively simple, and it does not need signal decomposition or complex variable transformation. Furthermore, the algorithm does not require a classifier for a separate training process, thus effectively reducing the chip area and operating power consumption to meet the conditions of implantable equipment. The brief delay characteristic of this circuit ensures that patients with epilepsy can receive timely electrical stimulation treatment during the onset of epilepsy. The results of this study are expected to be used in the study of EEG for real-time detection of epilepsy.

**Figure 13:**
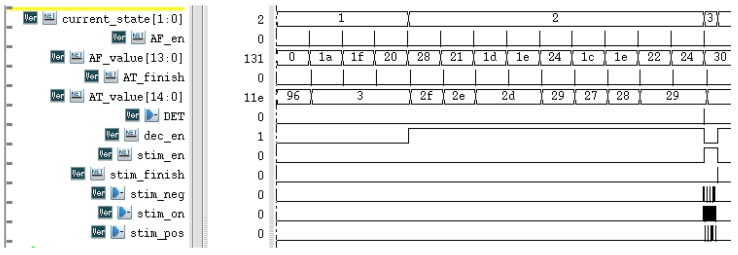
Simulation results of proposed circuit.

Simulation results are shown in Figure 13. The amplitude feature is taken as an example. First, in the threshold calculation, after four features are completely calculated, a new threshold AF_en is generated, and the state machine enters the feature detection step from the threshold calculation. The threshold and feature values AT_value and AF_value are constantly updated. When the detection module calculates that epilepsy has occurred, the DET signal is increased. The system enters stimulation mode (i.e., stim_en converts to 1), and the thresholds and features are no longer updated at this stage. The system then sends a control signal (stim_neg, stim_on, stim_pos, and stim_finish of the biphasic excitation current) to the analog part.

### 4.4. Digital Analog Hybrid Simulation

**Figure 14:**
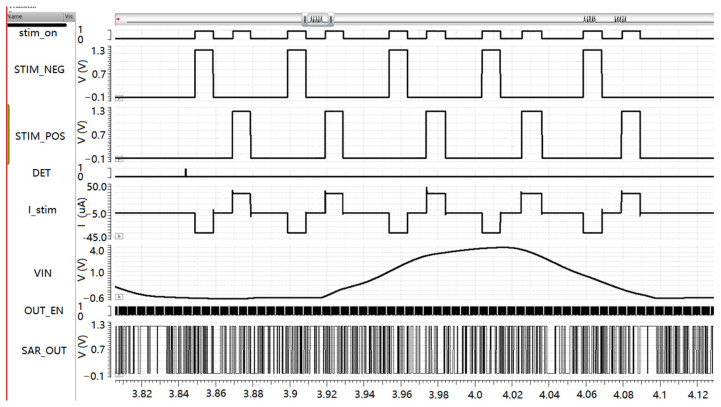
Digital analog hybrid simulation.

The AMS simulator was used for a digital and analog hybrid simulation. The input endpoint of the digital circuit is connected to the output endpoint of the SAR ADC. The data must be outputted in accordance with the output format of the ADC as a test incentive. The data are first stored in a register and then converted into a serial output signal SAR_OUT. The DET signal is a signal that detects a seizure. Two output signals—STIM_POS and STIM_NEG—representing the positive and negative phases of the pulse stimulus are present because it is a two-phase pulse current. The I_stim signal is the voltage that stimulates the output of the circuit. As shown in Figure 14, under the control of the positive and negative phases of the pulse stimulation, the output current is 34 μA.

## 5. Discussion

With the aim of improving on the disadvantages of existing open-loop and closed-loop nerve stimulation methods, a novel closed-loop epilepsy detection and stimulation structure is proposed in this study. First, AMP_FV, SLP_FV, LL_FV and POWER_FV are selected as features, and a threshold calculation algorithm that can be automatically updated is proposed. The resulting digital circuit has a power consumption and chip area of 0.8 μW and 0.023 mm^2^ only, respectively. The accuracy of detection is more than 94%. These characteristics are critical for implantable medical devices because they ensure an extended battery life and reduced physical impact on patients.

Compared with machine learning and deep learning algorithms, the four-feature computation and updatable threshold detection methods proposed in this paper do not require a long training process and complex training sets, making the circuit structure simpler than most existing closed-loop stimulation methods and more suitable for the implementation of invasive devices. Unlike open-loop nerve stimulation, which fixes stimulation parameters and stimulation time, this system can detect and treat patients in real time, a key advantage that allows the system to adapt to a patient’s changing physiology without manual intervention.

## 6. Conclusions

This study presents a closed-loop epilepsy detection system that integrates neural signal collection and epilepsy detection and stimulation. An effective time domain detection algorithm is proposed. The threshold is obtained by combining the mean and variance of four adjacent eigenvalues AMP_FV, POWER_FV, SLP_FV, and LL_FV. The validity of the time-domain algorithm to obtain the optimal threshold combination parameters is verified by the programming tool Python. The digital design of the epilepsy detection system is presented, including feature calculation module, threshold calculation module, feature detection module, stimulation control circuit, state control circuit, and ADC/SPI interface. Finally, the simulator tool AMS is used to simulate the digital and analog mixture, and the correct biphasic stimulation current output is obtained. With a power consumption of 0.8 μW and an area of 0.023 mm^2^, the system is 94% accurate and suitable for implantable devices. In the future, such devices could be fitted to people with epilepsy to provide alerts and treatment when seizures are detected, or even before they occur.

## Figures and Tables

**Table 1:** Four kinds of classification results.

		Prediction Class	
		Epileptic	Normal
**Real class**	**Epileptic**	TP	FN
	**Normal**	FP	TN

**Table 2:** The precision of the SLP parameter.

	M	1	2	3	4	5
N						
**1**		0.20	0.78	0.91	0.94	0.98
**2**		0.21	0.79	0.92	0.94	0.98
**3**		0.25	0.80	0.92	0.97	0.98
**4**		0.32	0.80	0.91	0.96	0.98
**5**		0.38	0.81	0.94	0.96	0.98

**Table 3:** The accuracy of the SLP parameter.

	M	1	2	3	4	5
N						
**1**		0.20	0.92	0.92	0.91	0.90
**2**		0.25	0.92	0.92	0.91	0.90
**3**		0.42	0.92	0.92	0.91	0.90
**4**		0.58	0.91	0.92	0.90	0.90
**5**		0.68	0.91	0.92	0.90	0.90

**Table 4:** The recall of the SLP parameter.

	M	1	2	3	4	5
N						
**1**		1.00	0.83	0.68	0.61	0.50
**2**		0.99	0.80	0.67	0.59	0.50
**3**		0.99	0.79	0.67	0.57	0.50
**4**		0.97	0.77	0.64	0.55	0.50
**5**		0.95	0.77	0.64	0.55	0.49

**Table 5:** The F2 Score of the SLP parameter.

	M	1	2	3	4	5
N						
**1**		0.56	0.82	0.72	0.66	0.55
**2**		0.57	0.80	0.71	0.64	0.55
**3**		0.63	0.79	0.71	0.62	0.55
**4**		0.69	0.78	0.68	0.60	0.55
**5**		0.73	0.78	0.68	0.6	0.54

**Table 6:** Optimal threshold parameters.

Feature	AMP	SLP	POWER	LL
M	5	2	3	2
N	4	1	1	1

**Table 7:** Comparison with previous work on FPGA platform.

Parameter	[34]	[35]	[36]	[37]	This Work
**Platform**	Xilinx Kintex-7 XC7K325T	Xilinx Zynq XC7Z030	Xilinx Artix-7 XC7A35T	Xilinx PYNQ-Z2 XC7Z020	Altera DE2-115
**Frequency**	100 MHz	100 MHz	180 MHz	100 MHz	200 KHz
**Power**	64 mw	N/A	82.589 mw	3.1 w	75 mw
**LUTs**	1737	5202	1158	1364	1095
**DSPs**	6	N/A	16	5	9

**Table 8:** Comparison of the proposed epilepsy-detection-stimulation circuit with previous works.

Parameters	[38]	[39]	[40]	[41]	This Work
**Process (nm)**	65	180	40	65	55
**ACC (%)**	87.9	88.73	86	91.86	94
**Chip Area (mm^2^)**	2.55	13.67	0.114	0.98	0.023
**Power Consumption (mW)**	4.45	0.992	0.311	0.42	0.8 × 10^−3^
**Classifier**	CNN	LLS	GTCA-SVM	SVM	Threshold
**Stimulation current (µA)**	N/A	N/A	N/A	N/A	34

## Data Availability

Epileptic EEG data sourced from an open dataset which can be downloaded at https://www.upf.edu/web/ntsa/downloads/ (accessed on 20 April 2024).

## Abbreviations

The following abbreviations are used in this manuscript:

EEG	Electroencephalogram
ADC	Analog to digital converter
DAC	Digital to analog converter
LDO	Low dropout regulator
SPI	Serial peripheral interface
LNA	Low noise amplifier
LPF	Low-pass filter
SAR	Successive approximation
AMP	Amplitude
SLP	Signal slope
LL	Line length
POWER	Energy
FIFO	First input first output
AVE	Mean value
STD	Standard deviation value
TP	True positive
TN	True negative
FP	False positive
FN	False negative
PW	Pulse width
FPGA	Field programmable gate array
LUT	Look-up tables
DSP	Digital signal processor
ASIC	Application-specific integrated circuits
CNN	Convolutional neural network
LLS	Linear Least Squares
TH_FINISH	Threshold calculation finish signal
